# Multi-group diagnostic classification of high-dimensional data using differential scanning calorimetry plasma thermograms

**DOI:** 10.1371/journal.pone.0220765

**Published:** 2019-08-20

**Authors:** Shesh N. Rai, Sudhir Srivastava, Jianmin Pan, Xiaoyong Wu, Somesh P. Rai, Chongkham S. Mekmaysy, Lynn DeLeeuw, Jonathan B. Chaires, Nichola C. Garbett

**Affiliations:** 1 Biostatistics and Bioinformatics Facility, James Graham Brown Cancer Center, University of Louisville, Louisville, Kentucky, United States of America; 2 Department of Bioinformatics and Biostatistics, University of Louisville, Louisville, Kentucky, United States of America; 3 Centre for Agricultural Bioinformatics, ICAR-Indian Agricultural Statistics Research Institute, New Delhi, India; 4 School of Public Health and Information Sciences, University of Louisville, Louisville, Kentucky, United States of America; 5 Department of Medicine, University of Louisville, Louisville, Kentucky, United States of America; 6 Biophysical Core Facility, James Graham Brown Cancer Center, University of Louisville, Louisville, Kentucky, United States of America; Universidad de Granada, SPAIN

## Abstract

The thermoanalytical technique differential scanning calorimetry (DSC) has been applied to characterize protein denaturation patterns (thermograms) in blood plasma samples and relate these to a subject’s health status. The analysis and classification of thermograms is challenging because of the high-dimensionality of the dataset. There are various methods for group classification using high-dimensional data sets; however, the impact of using high-dimensional data sets for cancer classification has been poorly understood. In the present article, we proposed a statistical approach for data reduction and a parametric method (PM) for modeling of high-dimensional data sets for two- and three- group classification using DSC and demographic data. We compared the PM to the non-parametric classification method K-nearest neighbors (KNN) and the semi-parametric classification method KNN with dynamic time warping (DTW). We evaluated the performance of these methods for multiple two-group classifications: (i) normal versus cervical cancer, (ii) normal versus lung cancer, (iii) normal versus cancer (cervical + lung), (iv) lung cancer versus cervical cancer as well as for three-group classification: normal versus cervical cancer versus lung cancer. In general, performance for two-group classification was high whereas three-group classification was more challenging, with all three methods predicting normal samples more accurately than cancer samples. Moreover, specificity of the PM method was mostly higher or the same as KNN and DTW-KNN with lower sensitivity. The performance of KNN and DTW-KNN decreased with the inclusion of demographic data, whereas similar performance was observed for the PM which could be explained by the fact that the PM uses fewer parameters as compared to KNN and DTW-KNN methods and is thus less susceptible to the risk of overfitting. More importantly the accuracy of the PM can be increased by using a greater number of quantile data points and by the inclusion of additional demographic and clinical data, providing a substantial advantage over KNN and DTW-KNN methods.

## Introduction

Differential scanning calorimetry (DSC) analysis of human blood plasma has been used as a method to detect disease related changes in the plasma proteome [1–6]. DSC analysis is a thermoanalytical technique that measures how the physical properties of biomolecule solutions change with temperature. Specifically, DSC precisely measures heat capacity changes as a function of temperature, yielding a profile known as a thermogram, which is specific for a given biomolecule. As heat capacity is an extensive property, the DSC thermogram is extremely sensitive to the precise composition of biomolecule mixtures with the observed signal related to the amount, interaction or modification of component biomolecules. It is this characteristic of DSC thermograms, to reflect disease-related changes in the blood plasma proteome, which forms the basis of the utility of DSC as a novel diagnostic technology. For healthy normal individuals, the DSC thermogram reflects the sum of the thermograms of component proteins weighted according to their normal abundance in plasma [7]. Plasma thermograms from patients suffering from a variety of diseases appear different in amplitude and denaturation temperature [3–5, 8–14]. Preliminary data show that these differences correlate with the type and stage of disease; we hypothesize that these are related to disease-specific changes in the concentration, modification or intermolecular interactions of components within the plasma proteome. The alteration of the plasma thermogram of diseased individuals relative to that of normal individuals would make qualitative identification of disease status trivial. However, thermogram changes are complex, making disease classification a challenging task [3, 8, 15–18].

We have previously described statistical approaches for the classification of plasma thermograms according to disease status, an essential step in the development of the clinical utility of DSC thermograms [16]. In this previous work we proposed a two-group classification method using only DSC data and compared our method to the I-RELIEF method [19, 20]. Our approach used a parametric method based on a linear model whereas the I-RELIEF method is an adaptation of a nonparametric method. We performed two-group classification for normal and cervical cancer samples and found that our method performed better than the I-RELIEF method [16]. The objective of our current study is to classify subjects between control and multiple case groups based on the characteristics of thermogram data sets. We have extended our previous work [16] to include the incorporation of demographic information in our classification method involving two groups (normal and diseased) as well as applying the classification method to three groups (normal and two disease states). The performance of the method was demonstrated through its application to three thermogram data sets: 1) commercially obtained plasma samples from healthy normal individuals, 2) plasma specimens obtained from patients having cervical cancer, 3) plasma specimens obtained from lung cancer patients. We also compared our method with two commonly used classification approaches, the non-parametric approach KNN and the semi-parametric approach DTW-KNN.

## Materials and methods

### Characteristics of plasma samples

Plasma samples from 100 healthy individuals with demographic characteristics were purchased from Innovative Research (Southfield, MI). Cervical cancer specimens were obtained from women attending the clinics of the Division of Gynecologic Oncology with invasive cervical carcinoma. Lung cancer specimens were obtained from patients attending the clinics of the Division of Thoracic Oncology. The study protocol and patient consent procedures were approved by the University of Louisville Institutional Review Board (IRB# 08.0108, 08.0636, 608.03, 08.0388). All patients gave written informed consent for their blood and tissues to be entered into a tissue repository (IRB# 608.03, 08.0388) and utilized for research purposes. The IRB specifically approved the use of plasma specimens from the bio repository for use in this study without the need for further consent (IRB# 08.0108, 08.0636). All specimens collected for the study were deidentified. Associated demographic and clinical information was collected by clinical trials office personnel and securely stored on the bio repository computer. The bio repository was approved by the University of Louisville Institutional Review Board (IRB# 608.03, 08.0388) and was fully HIPAA compliant. Specimens provided for DSC studies were coded by bio repository collection number. In this form, specimens were deidentified and blinded for demographic and pathologic disease status for unbiased data collection. Demographic and clinical status was subsequently provided for data analysis. Blood was drawn into 6 mL green top (plasma; sodium heparin anticoagulant) vacutainers. Tubes were gently mixed by inversion 8–10 times immediately after blood collection to evenly distribute the anticoagulant additive followed by centrifugation at 3200 rpm for 10 min (BD-Clay Adams Compact II centrifuge). Separated plasma was carefully aspirated to avoid hemolysis or contamination of the separated blood phases, aliquoted and immediately stored at −80 °C until analysis. All handling of specimens and specimen waste was in accordance with OSHA bloodborne pathogen procedures.

### Collection of DSC thermogram data

Plasma samples were prepared for DSC analysis according to our previously published method [2, 21]. Briefly, samples were dialyzed against a standard phosphate buffer (1.7 mM KH_2_PO_4_, 8.3 mM K_2_HPO_4_, 150 mM NaCl, 15 mM sodium citrate, pH 7.5) for 24 h at 4 °C to achieve normalization of buffer conditions for all samples obtained in different plasma anticoagulants. Samples were diluted 25-fold with dialysate to obtain a suitable concentration for DSC analysis. DSC data were collected using a VP-Capillary automated DSC instrument (MicroCal, now Malvern Pananalytical, Northampton, MA). Samples and dialysate were stored in 96-well plates and transferred to the instrument autosampler thermostated at 5 °C until being loaded into the calorimeter by the robotic attachment. DSC scans were recorded from 20 °C to 110 °C at 1 °C/min using the mid feedback mode, a filtering period of 2 s and a prescan thermostat of 15 min. Total protein concentrations of plasma samples for normalization of DSC data were measured using the bicinchoninic acid protein assay kit microplate procedure (Pierce, Rockford, IL), with minor modifications to the manufacturer’s protocol. DSC data were processed using Origin version 7 (OriginLab Corporation, Northampton, MA). The process was as follows: 1) correct for the instrument baseline by subtracting an appropriate buffer reference scan, 2) normalize for the total gram concentration of protein in each sample, 3) fit for nonzero sample baselines by applying a linear baseline function. Final thermogram data were the average of duplicate measurements and plotted as excess specific heat capacity (cal/°C.g) versus temperature (°C). Examination of thermogram data revealed that the temperature range 45–90°C spanned the denaturation profile for all samples and scans were truncated to this range for subsequent analyses. Three control samples were flagged as poor quality data and removed prior to analysis.

### Experimental data and dimensionality reduction

**Fig 1:**
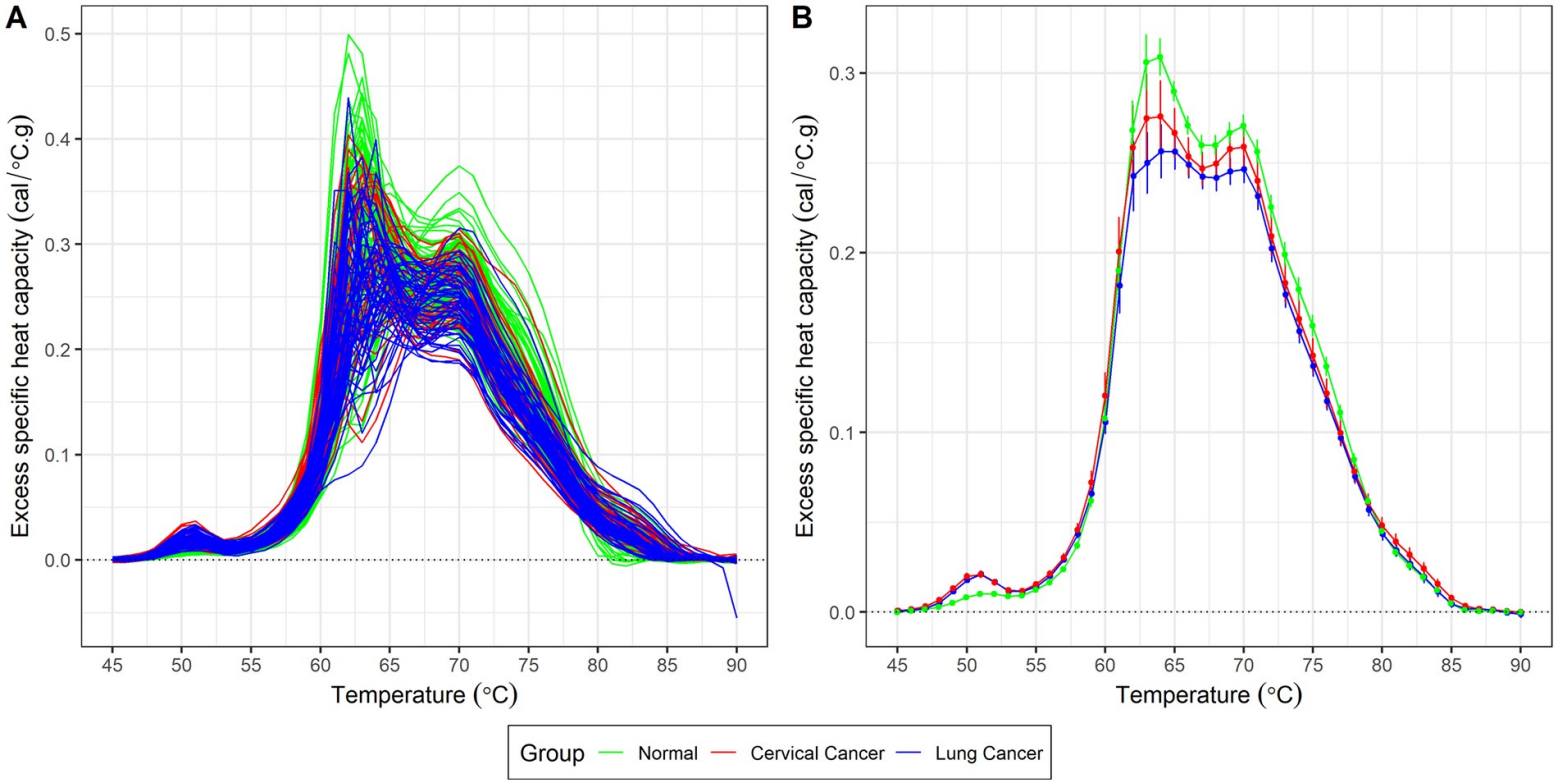
The composite line plot and error bar plot. (A) Composite line plot of HC values at each temperature data point for 97 normal (green), 35 cervical cancer (red) and 54 lung cancer (blue) samples. (B) Composite error bar plot of HC values at each temperature data point for three groups: normal (green), cervical cancer (red) and lung cancer (blue). The circles represent mean values and the error bars represent the 95% confidence interval.

The DSC thermogram data set is comprised of heat capacity (HC) values at 0.1°C intervals over the temperature range 45°C-90°C for a total of 186 samples from three clinical groups: (i) normal/ control (*C*_*0*_) [97 samples], (ii) cervical cancer (*CC*) [35 samples], (iii) lung cancer (*LC*) [54 samples]. There are 451 data points per thermogram with each sample having duplicate measurements. We take the mean of the duplicate measurements to get a single measurement at each of these data points for each sample (S1 File). The composite line plots of HC values with error bars (95% confidence interval) at each temperature point for three groups are shown in Fig 1.

We reduced the high-dimensionality of the data by taking the mean of HC values within each 1°C temperature increment. For example, we take the mean of HC values of a sample at 45°C, 45.1°C, …, 45.8°C, 45.9°C to get the mean HC value at the temperature data point 45°C. Thus, we have mean HC values at 45°C, 46°C, …, 89°C, 90°C for each sample. Furthermore, on examining the data, we observed baseline fluctuation in the HC signal at the low temperature and high temperature regions of the thermograms resulting in negative HC values below 48°C and above 80°C in many samples. Negative input values are incompatible with the parametric model; therefore, we truncated the thermograms to the temperature range 48°C to 80°C (a total of 33 data points) for all samples.

### Demographic data for two- and three-group classification

We have incorporated demographic data (age, ethnicity and gender) of the individuals for the development of the classification method (S1 File). Differences between the clinical groups among demographic factors are detailed in Table 1. The age (in years) of individuals is similar for the *C*_*0*_ and *CC* groups with a higher age range associated with the *LC* group. The observations for the *CC* and *LC* groups are consistent with the demographic characteristics of the patient populations seen at the James Graham Brown Cancer Center clinics. There are two levels in the variables, ethnicity [White (Non-Hispanic or Latino) or others (African-American, or White (Hispanic or Latino)] and gender [male or female].

**Table 1:** Demographic, clinical and data characteristics of the study group.

	Control N (%)	Cervical cancer N (%)	Lung cancer N (%)
**Demographic characteristics**			
**Gender**			
Male	50 (51.5)	N / A	22 (40.7)
Female	47 (48.5)	35 (100)	32 (59.3)
**Ethnicity / Race**			
African-American	20 (20.6)	4 (11.4)	14 (25.9)
White			
Non-Hispanic or Latino	50 (51.5)	28 (80.0)	40 (74.1)
Hispanic or Latino	27 (27.8)	3 (8.6)	0 (0)
**Age**			
Range (years)	18–61	26–66	42–86
Age (years) mean (sd)	35.8 (11.2)	46.5 (11.8)	61.8 (11.6)
**Clinical characteristics**			
**Stage**			
I	N / A	14	2
II	N / A	10	3
III	N / A	7	15
IV	N / A	4	30
Limited	N / A	N / A	2
Not Staged	N / A	N / A	2
**Data set characteristics**			
**Thermogram data points per sample**			
Original data: 45°C -90°C; 0.1°C intervals; duplicate scans	902	902	902
Truncated, averaged data: 48°C -80°C; 1°C intervals; averaged scans	33	33	33
**Total number of samples per group**	97	35	54
**Total data points per group**			
Original data: 45°C -90°C; 0.1°C intervals; duplicate scans	87,494	31,570	48,708
Truncated, averaged data: 48°C -80°C; 1°C intervals; averaged scans	3,201	1,155	1,782

### Normality test based on different transformation methods

We applied the Shapiro-Wilk normality test to check the assumption of normality at each temperature data point of the combined data obtained from the three groups as well as pairwise two groups (*C*_*0*_ and *CC*, *C*_*0*_ and *LC*, *CC* and *LC*) [22, 23]. The following transformations were evaluated:

$$H_{1}=\text{log}\left(H\right),\;H_{2}=logit(H/0.5),\;H_{3}=\frac{e^{H}}{1+e^{H}},\;H_{4}=\frac{e^{2H}}{1+e^{2H}}$$

where *H* is the original data. The null hypothesis is that the data are normally distributed at each temperature data point. We have chosen the level of significance as 0.05. If the p-value is less than 0.05, then the null hypothesis that the data are normally distributed is rejected. If the p-value is greater than 0.05, then the null hypothesis is not rejected, i.e., the data are normally distributed. For each transformation, we calculated the probability of p-values greater than 0.05 for *K* temperature points $\left(P=\frac{\#\;of\;\{p>0.0.5\}}{K}\right)$. Transformations with the maximum number of temperature points following a normal distribution were selected for development of the classification method.

### Parametric method of data classification

#### Model selection

After selecting the appropriate transformation, we fit the regression model as given below:

$$Y=\alpha +\beta^{T}X+\varepsilon$$ (1)

Here, *Y* is the response variable (HC values) and the covariate *X* includes different temperatures (*T*), the group variable *G*, the interaction *T* × *G* and demographic data (age, gender and ethnicity). A 4- degree polynomial for different temperatures has been used [*T*_1_ = (*T*–*mean*(*T*))/*sd*(*T*), $T_{i}=T_{1}^{i}\;(i=2,\;3,\;4)]$. We have also tried other degrees of polynomial (2-, 3-, 5- and 6- degree). However, we got the best classification results using a 4-degree polynomial. The variable *G* is the group indicator: 0 for control and 1 for case in two-group classification; 0 for control, 1 for case 1 and 2 for case 2 group in three-group classification. The intercept term *α* and the vector of coefficients *β*^*T*^ are the unknown parameters to be estimated. The last term *ε* is the random error component. We use all the observations from the samples based on the data for two group and three group classification methods for model selection. The model selection is based on Akaike information criterion (AIC) in a stepwise algorithm [24, 25]. We perform a stepwise regression to select important variables from a set of explanatory variables. At each step, a variable is considered for addition or subtraction based on the AIC value and the selected models were the ones having the lowest AIC value. The details of the models selected are provided in the Results section.

#### Two-group classification method

We have developed a two-group classification method based on previous work [16] with some modifications. Suppose there are *n*_0_ control samples and *n*_1_ case samples, where *n*_0_ + *n*_1_ = *N*. There are *K* temperature data points for each sample. Let *Y* and *X* be the response variable and covariate vector, respectively, and *G* be the group indicator, 0 for control and 1 for case. The steps involved are given below:

1 **Computation of *p*% quantile of residuals**: We compute the *p*% quantile of residuals, *e*.*g*., 95% quantile, at each point using *N* observations from all the samples.
  1.1 Fit the selected regression model as given in the previous section using all the measurements from *N* samples.
  1.2 Calculate the residuals for all the *N* × *K* observations, denoted as *R*_*i*,*j*_(*i* = 1, 2, …, *K and j* = 1, 2, …, *N*). The residual (*R*_*i*,*j*_) is the difference between the observed value of dependent variable (*Y*_*i*,*j*_) and the predicted value ${(\hat{Y}}_{i,j})$, i.e., $R_{i,j}=Y_{i,j}{-\hat{Y}}_{i,j}$.
  1.3 Calculate the *p*% quantile of each of the *N* absolute values of residuals *R*_*i*,1_, *R*_*i*,2_, …, *R*_*i*,*N*_, for each temperature point *i* (*i* = 1, 2, …, *K*) denoted as *q*_*p*,1_, *q*_*p*,2_, …, *q*_*p*,*K*_.

In this paper, we have computed five different quantiles of residuals, *i*.*e*., 95%, 96%, 97%, 98% and 99% by using this step. We have used five different quantiles of residuals to get a more robust result. We have used the same data with all the samples as the reference data for model selection and computation of the quantile of residuals.

2 **Estimation of the parameters from the training dataset**: We randomly select *m*_0_ and *m*_1_ samples, respectively from *n*_0_ control samples and *n*_1_ case samples. Then, we use *m*_0_ + *m*_1_ samples to fit the regression model and estimate the parameters.
3 **Classification of samples in the testing dataset**: We validate the classification method using the remaining *n*_0_ − *m*_0_ control samples and *n*_1_ − *m*_1_ case samples.
  3.1 We compute the predicted observation for (*n*_0_ − *m*_0_ + *n*_1_ − *m*_1_) samples by using the estimate of parameters obtained from the previous step. We compute their prediction residuals for both *G* = 0 and 1. Therefore, there are 2*K* residuals for each sample, *r*_*i*_(*G* = 0) for *G* = 0 and *r*_*i*_(*G* = 1) for *G* = 1.
  3.2 We compare each *K* residuals to the quantiles derived in *Step 1* for *G* = 0 and *G* = 1, and calculate the *P*-values according to the Eq 2 given below, using each quantiles of residuals (p = 95%, 96%, 97%, 98% and 99%):
    $$P(G=j)=\frac{\left\{\#\;of\;|r_{i}(G=j)|\leq \;q_{p,i},\;i=1,\;2,\;\ldots ,\;K\right\}}{K}\;for\;j=0,\;1$$ (2)
  3.3 Classification is done by evaluating the following criteria:
    (i) If for a sample, *P*(*G* = 0) = *P*(*G* = 1), classify it to the neither of the two groups, and assign it a *NA* value. If there are no samples satisfying this condition, then go to next step.
    (ii) From the remaining samples, if *P*(*G* = 0) > *P*(*G* = 1) for a sample, classify it to the control group, otherwise classify it to the case group. Now, we have obtained five different classifications based on five different quantiles.
  3.4 Calculate the proportion of each group (case, control and group with *NA* values) for each sample based on classification using five different quantiles. If for a sample, the proportion of *NA* is 1, then randomly assign it to either control or case group. Then, from the remaining samples, if the proportion of case is more than that of control, then classify a sample to the case group and vice versa. If there is tie, then the tie is broken randomly.
  3.5 Compute the following accuracy measures [26]: sensitivity (*Sens*), specificity (*Spec*), positive predictive value (*PPV*), negative predictive value (*NPV*), accuracy (*Acc*) and balanced accuracy (*Bal Acc*) [S2 File].

#### Three group classification method

The above method has been extended to three group classification. Suppose there are three groups of samples: normal (control) with *n*_0_ samples, cervical cancer (case 1) with *n*_1_ samples and lung cancer (case 2) with *n*_2_ samples, where *n*_0_ + *n*_1_ + *n*_2_ = *N*. There are *K* measurement points (temperature) for each sample. Let *Y* and *X* be the response variable and covariate vector, respectively, and *G* be the group indicator, 0 for control, 1 for case 1 and 2 for case 2.

1 **Computation of *p*% quantile of residuals**: We use the same process as given in *step 1* of two-group classification method to compute the *p*% quantile of residuals at each point using *N* observations from all the samples.
2 **Estimation of the parameters from the training dataset**: We randomly select *m*_0_, *m*_1_ and *m*_2_ samples, respectively from *n*_0_ control samples, *n*_1_ case 1 samples and *n*_2_ case 2 samples. Then, use *m*_0_ + *m*_1_ + *m*_2_ samples to fit the regression model and estimate the parameters.
3 **Classification of samples in the testing dataset**: We validate the classification method using the remaining *n*_0_ − *m*_0_ control samples, *n*_1_ − *m*_1_ case 1 samples and *n*_2_ − *m*_2_ case 2 samples.
  3.1 Using the parameter estimates obtained in *step 2*, we compute the predicted observation for (*n*_0_ − *m*_0_ + *n*_1_ − *m*_1_ + *n*_2_ − *m*_2_) samples, and their prediction residuals for *G* = 0, 1, 2. We have 3*K* residuals for each sample data point *r*_*i*_(*G* = 0) for *G* = 0, *r*_*i*_(*G* = 1) for *G* = 1, and *r*_*i*_(*G* = 2) for *G* = 2.
  3.2 We compare each *K* residuals to the quantiles derived in *step 1* for *G* = 0, *G* = 1 and *G* = 2, and calculate the *P*-values using each quantiles of residuals according to Eq 3 below:
    $$P(G=j)=\frac{\left\{\#\;of\;|r_{i}(G=j)|\leq q_{p,i}\;,\;i=1,\;2,\;\ldots ,\;K\right\}}{K}\;for\;j=0,\;1,\;2$$ (3)
    Here, |*r*_*i*_(*G* = *j*)| is the absolute value of residuals.
  3.3 Classification is done by evaluating the following criteria:
    (i) Find the samples for which *P*(*G* = 0) = *P*(*G* = 1) = *P*(*G* = 2) and classify them to none of the three groups and assign *NA* values. If there are no samples satisfying this condition, then go to the next step.
    (ii) From the remaining samples, if for a sample *P*(*G* = 0) > *P*(*G* = 1) or *P*(*G* = 0) > *P*(*G* = 2), classify it to the control group otherwise classify it to a group G-12 having case 1 or case 2.
    (iii) In the group G-12, find the samples for which *P*(*G* = 1) = *P*(*G* = 2) and classify them to the neither of the two case groups, and assign NA values. If there are no samples satisfying this condition, then go to next step.
    (iv) From the remaining samples, if *P*(*G* = 1) > *P*(*G* = 2) for a sample, classify it to the case 1 group, otherwise classify it to the case 2 group. We have obtained five different classifications based on five different quantiles.
  3.4 Calculate the proportion of each group (control, case 1, case 2 and group with *NA* values) for each sample based on classification using five different quantiles. If for a sample, the proportion of *NA* is 1, then randomly assign either control or case 1 or case 2 group. Then, further classify the remaining samples by using these conditions: (i) if proportion of control is more than that of case 1 and case 2, then classify it to the control group; (ii) if proportion of case 1 is more than that of case 2 and control, then classify it to the case 1 group; (iii) if proportion of case 2 is more than that of case 1 and control, then classify it to the case 2 group. If there is a tie in any of these three conditions, then the tie is broken randomly.
  3.5 Compute the following accuracy measures for three-group classification: sensitivity (*Sens*), specificity (*Spec*), positive predictive value (*PPV*), negative predictive value (*NPV*), balanced accuracy (*Bal Acc*) and accuracy (*Acc*) [S2 File].

### Comparison of data classification methods

We have compared three different methods of classification: (i) Parametric method, (ii) KNN with DTW as distance function (DTW-KNN) [27–30] and (iii) KNN method [25, 27]. We have discussed the parametric method in the previous section. In DTW-KNN method, we have used the DTW distance [28] and then applied the KNN method for the classification. Here, the DTW distance is the distance obtained using the DTW approach to get the optimal alignment between two time series (example: a sample from the test dataset and a sample from the training dataset). Later we modified this method to calculate DTW distance with the inclusion of the demographic data for data classification. In the KNN method, for each sample of the test dataset, we use the Euclidean distance from the training dataset to find the *k* nearest samples. Then, the classification of the sample is decided by majority vote, with ties broken at random.

**Fig 2:**
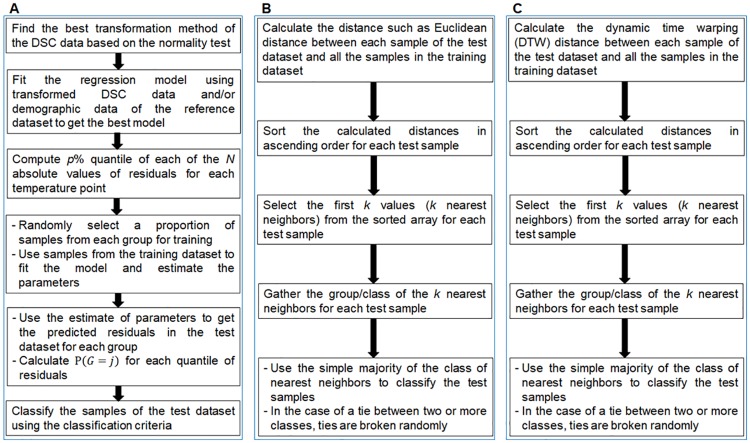
Overview of the classification methods. (A) Parametric method, (B) K-nearest neighbors method and (C) Dynamic time warping- K-nearest neighbors method.

Flowcharts of the main steps involved in the parametric method, KNN method and DTW-KNN method are shown in Fig 2 and S3 File.

The three classification approaches were applied to evaluate the discrimination of various two group combinations: (i) *C*_*0*_ vs. *CC*, (ii) *C*_*0*_ vs. *LC*, (iii) *CC* vs. *LC* and (iv) *C*_*0*_ vs. (*CC*+*LC*) as well as the classification of all three groups [*C*_*0*_ vs. *CC* vs. *LC*]. We evaluated classification performance using only DSC data as well as combining DSC data and demographic data and compared the performance of our developed method with the semi-parametric method DTW-KNN and the non-parametric method KNN. We calculated accuracy measures for each classification [26]. To assess variability in classification performance we repeated each method 500 times to get the average values and standard deviation of each accuracy measure. We implemented the methods and completed all the analysis in the statistical analysis program, R [29] (S6 File).

## Results

### Selection of the transformation method

Table 2 shows p-values of the normality test at each temperature data point for each transformation of the combined data from three groups. Results of the normality test for data for each pairwise combination of groups are shown in S4 File.

**Table 2:** Results of the normality test showing p-values at different temperature points using data transformations for three-group classification.

Temperature (°C)	*H*	*H*_*1*_	*H*_*2*_	*H*_*3*_	*H*_*4*_
48	0	0.01	0.01	0	0
49	0	0	0	0	0
50	0	0	0	0	0
51	0	0.01	0.01	0	0
52	0	0.05	0.05	0	0
53	0	0.11	0.13	0	0
54	0	0.64	0.66	0	0
55	0	0.35	0.37	0	0
56	0	0.57	0.57	0	0
57	0	0.76	0.77	0	0
58	0	0.47	0.47	0	0
59	0	0.34	0.3	0	0
60	0	0.04	0.02	0	0
61	0	0.28	0.13	0	0
62	0.01	0.12	0.72	0.01	0.03
63	0.86	0	0.02	0.86	0.8
64	0.08	0	0	0.06	0.02
65	0.02	0	0	0.01	0.01
66	0.61	0.77	0.88	0.64	0.72
67	0.15	0.75	0.63	0.17	0.22
68	0.2	0.65	0.63	0.22	0.29
69	0.5	0.71	0.84	0.54	0.65
70	0.54	0.7	0.87	0.58	0.7
71	0.29	0.8	0.86	0.33	0.45
72	0.07	0.91	0.87	0.09	0.14
73	0	0.85	0.53	0	0
74	0	0.25	0.06	0	0
75	0	0.03	0	0	0
76	0	0.02	0	0	0
77	0	0.07	0.02	0	0
78	0	0.31	0.17	0	0
79	0	0.74	0.77	0	0
80	0.18	0	0	0.18	0.19
**Mean**	0.11	0.34	0.34	0.11	0.13
**No. of *P* > 0.05**	10	21	21	10	9
**% of *P* > 0.05**	30.3	**63.64**	**63.64**	30.3	27.27

Legend: *H*_1_ = log(*H*), *H*_2_ = *logit*(*H*/0.5), $H_{3}=\frac{e^{H}}{1+e^{H}},\;H_{4}=\frac{e^{2H}}{1+e^{2H}}$

The result given in Table 2 shows that both the transformations *H*_1_ and *H*_2_ can be selected based on the p-value. Tables A and B in S4 File show that the transformation *H*_1_ is the best for both *C*_*0*_ vs. *LC* and *CC* vs. *LC*. Table C in S4 File shows transformationn *H*_2_ is slightly better than *H*_1_ for *CC* vs. *LC*. We have also performed principal components analysis using the original data (*H*) and log transformed data (*H*_1_) (S1 Fig). We found that the samples for the three groups are overlapped for the original data but the normal group is more separable from the samples of the cancer group (*CC* and *LC*) in case of the log transformed (*H*_1_) data. Therefore, we have selected the most common transformation, i.e., the logarithmic transformation (*H*_1_) for further analysis.

### Selection of the best regression model

All the observations from the samples were fitted based on the data for two group and three group classification methods. The regression models using DSC data alone and combining DSC and demographic data, respectively, are given by Eqs (4) and (5).

$$H_{1}=\alpha +\beta_{1}T_{1}+\beta_{2}T_{2}+\beta_{3}T_{3}+\beta_{4}T_{4}+\beta_{5}G+\beta_{6}T_{1}G+\beta_{7}T_{2}G+\beta_{8}T_{3}G+\beta_{9}T_{4}G+\varepsilon$$ (4)

$$H_{1}=\alpha +\beta_{1}T_{1}+\beta_{2}T_{2}+\beta_{3}T_{3}+\beta_{4}T_{4}+\beta_{5}G+\beta_{6}T_{1}G+\beta_{7}T_{2}G+\beta_{8}T_{3}G+\beta_{9}T_{4}G+\beta_{10}Age+\beta_{11}Ethnicity+\beta_{12}Gender+\varepsilon$$ (5)

where, *H*_1_ = log(*H*), *T*_1_ = (*T* − *mean*(*T*))/*sd*(*T*), $T_{i}=T_{1}^{i}\;(i=2,\;3,\;4)$, *G* is the group indicator (0 for control and 1 for case in two-group classification; 0 for control, 1 for case 1 and 2 for case 2 group in three-group classification) for both Eqs (4) and (5). The terms “Age”, “Gender” and “Ethnicity” in Eq (5) correspond to the demographic data of the subjects. Various models were evaluated with the inclusion of different demographic variables and the best performing models were selected for each classification based on the transformed data in Table 3. As the age distribution in the control group is different to that in the cervical cancer and lung cancer groups, we evaluated the effect of age on thermogram profiles (S5 File). We divided the control group into two age groups using a median cut-off of 36 years (age ≤ 36 years and age > 36 years) and compared thermogram data at each temperature point using a two-sample t-test. The test was found to be significant (p-value < 0.05) for only three temperature points (78 °C, 79 °C and 80 °C) out of a total of 33 points. Adjusting the p-values for multiple comparisons using the “bonferroni” method, we found the test to be significant at only one temperature point (79 °C). Furthermore, age was not a significant variable in demographic models for both two- and three-group classification.

**Table 3:** Transformation and model selected for different classifications.

Data used	Classification	Model selected
DSC data	*C*_*0*_ *vs*. *CC*	H1 ~ T1 + T2 + T3 + T4 + G + T1:G + T2:G + T3:G + T4:G
	*C*_*0*_ *vs*. *LC*	H1 ~ T1 + T2 + T3 + T4 + G + T1:G + T2:G + T3:G
	*C*_*0*_ *vs*. *(CC+LC)*	H1 ~ T1 + T2 + T3 + T4 + G + T1:G + T2:G + T3:G
	*CC vs*. *LC*	H1 ~ T1 + T2 + T3 + T4 + G + T2:G + T3:G + T4:G
	*C*_*0*_ *vs*. *CC vs*. *LC*	H1 ~ T1 + T2 + T3 + T4 + G + T1:G + T2:G + T3:G
DSC + demographic data	*C*_*0*_ *vs*. *CC*	H1 ~ T1 + T2 + T3 + T4 + G + T1:G + T2:G + T3:G + T4:G + Ethnicity + Gender
	*C*_*0*_ *vs*. *LC*	H1 ~ T1 + T2 + T3 + T4 + G + T1:G + T2:G + T3:G + Ethnicity + Gender
	*C*_*0*_ *vs*. *(CC+LC)*	H1 ~ T1 + T2 + T3 + T4 + G + T1:G + T2:G + T3:G + Ethnicity + Gender
	*CC vs*. *LC*	H1 ~ T1 + T2 + T3 + T4 + G + T2:G + T3:G + T4:G
	*C*_*0*_ *vs*. *CC vs*. *LC*	H1 ~ T1 + T2 + T3 + T4 + G + T1:G + T2:G + T3:G + Ethnicity

Normal/ control (*C*_*0*_), cervical cancer (*CC*), and lung cancer (*LC*). See Eqs 4 and 5 for definition of the other terms

### Validation results

We have used 70% of the samples as the training dataset for the two group and three group classification methods. We have 68 normal, 24 cervical cancer, 38 lung cancer samples and 62 cancer (*CC* + *LC*) samples in the training dataset. The remaining 29 normal, 11 cervical cancer, 16 lung cancer samples and 27 cancer (*CC* + *LC*) samples have been used as the testing dataset.

**Results of our proposed parametric method and comparison with commonly used semi-parametric and non-parametric classification methods**: Results of various two groups classification using different classification methods based on DSC data and DSC data including demographic data are presented in Table 4.

**Table 4:** Results of two group classification methods.

	Groups	Methods	*Acc*	*Sens*	*Spec*	*PPV*	*NPV*	*Bal Acc*
**DSC data**	***C***_***0***_ ***vs*. *CC***	**PM**	0.86 (0.05)	0.83 (0.07)	0.94 (0.06)	0.97 (0.03)	0.69 (0.10)	0.89 (0.04)
		**KNN**	0.96 (0.03)	0.99 (0.02)	0.86 (0.10)	0.95 (0.03)	0.98 (0.04)	0.93 (0.05)
		**DTW-KNN**	0.96 (0.03)	1.00 (0.00)	0.84 (0.10)	0.95 (0.03)	1.00 (0.01)	0.92 (0.05)
	***C***_***0***_ ***vs*. *LC***	**PM**	0.85 (0.06)	0.82 (0.08)	0.91 (0.07)	0.94 (0.04)	0.74 (0.08)	0.86 (0.05)
		**KNN**	0.94 (0.03)	0.97 (0.03)	0.90 (0.08)	0.95 (0.04)	0.94 (0.05)	0.93 (0.04)
		**DTW-KNN**	0.94 (0.03)	0.96 (0.03)	0.91 (0.06)	0.95 (0.03)	0.94 (0.06)	0.94 (0.03)
	***CC vs*. *LC***	**PM**	0.59 (0.08)	0.54 (0.13)	0.62 (0.12)	0.50 (0.10)	0.66 (0.07)	0.58 (0.08)
		**KNN**	0.69 (0.08)	0.43 (0.15)	0.87 (0.09)	0.72 (0.17)	0.69 (0.06)	0.65 (0.08)
		**DTW-KNN**	0.68 (0.07)	0.38 (0.14)	0.89 (0.08)	0.73 (0.17)	0.68 (0.05)	0.64 (0.07)
	***C***_***0***_ ***vs*. *CC+LC***	**PM**	0.86 (0.05)	0.81 (0.07)	0.90 (0.05)	0.90 (0.05)	0.82 (0.06)	0.86 (0.04)
		**KNN**	0.94 (0.03)	0.96 (0.03)	0.92 (0.05)	0.93 (0.04)	0.96 (0.04)	0.94 (0.03)
		**DTW-KNN**	0.93 (0.03)	0.95 (0.04)	0.92 (0.05)	0.93 (0.04)	0.94 (0.04)	0.93 (0.03)
**DSC and demographic data**	***C***_***0***_ ***vs*. *CC***	**PM**	0.85 (0.05)	0.82 (0.07)	0.93 (0.06)	0.97 (0.03)	0.68 (0.09)	0.88 (0.05)
		**KNN**	0.83 (0.05)	0.96 (0.04)	0.49 (0.14)	0.84 (0.04)	0.86 (0.14)	0.73 (0.07)
		**DTW-KNN**	0.89 (0.05)	0.99 (0.02)	0.63 (0.16)	0.88 (0.05)	0.96 (0.07)	0.81 (0.08)
	***C***_***0***_ ***vs*. *LC***	**PM**	0.85 (0.05)	0.81 (0.07)	0.91 (0.07)	0.94 (0.04)	0.74 (0.08)	0.86 (0.05)
		**KNN**	0.89 (0.04)	0.91 (0.06)	0.86 (0.08)	0.92 (0.04)	0.84 (0.08)	0.88 (0.04)
		**DTW-KNN**	0.81 (0.06)	1.00 (0.01)	0.47 (0.16)	0.78 (0.05)	0.99 (0.02)	0.73 (0.08)
	***CC vs*. *LC***	**PM**	0.59 (0.08)	0.54 (0.13)	0.62 (0.12)	0.50 (0.10)	0.66 (0.07)	0.58 (0.08)
		**KNN**	0.77 (0.06)	0.60 (0.15)	0.89 (0.08)	0.81 (0.12)	0.77 (0.07)	0.75 (0.07)
		**DTW-KNN**	0.67 (0.01)	0.80 (0.10)	0.57 (0.15)	0.58 (0.10)	0.81 (0.10)	0.69 (0.09)
	***C***_***0***_ ***vs*. *CC+LC***	**PM**	0.86 (0.04)	0.81 (0.07)	0.91 (0.05)	0.91 (0.05)	0.82 (0.06)	0.86 (0.04)
		**KNN**	0.84 (0.04)	0.88 (0.06)	0.81 (0.08)	0.84 (0.06)	0.86 (0.06)	0.84 (0.05)
		**DTW-KNN**	0.87 (0.04)	0.98 (0.03)	0.76 (0.09)	0.82 (0.06)	0.97 (0.03)	0.87 (0.05)

**Note**: The accuracy measures are denoted by accuracy (*Acc*), sensitivity (*Sens*), specificity (*Spec*), positive predictive value (*PPV*), negative predictive value (*NPV*) and balanced accuracy (*Bal Acc*). The groups are denoted by normal/ control (*C*_*0*_), cervical cancer (*CC*) and lung cancer (*LC*). The methods of classifications used are our parametric or proposed method (PM), KNN and DTW-KNN. Mean values of accuracy measures are shown with standard deviation in parentheses. Mean values less than 50 are shaded in red, values 50–84 are shaded in grey and values greater than or equal to 85 are shaded in green.

Results of three group classification based on DSC data alone and combined DSC and demographic data are shown in Table 5.

**Table 5:** Results of three group classification methods.

	Methods	Groups	*Sens*	*Spec*	*PPV*	*NPV*	*Bal Acc*	*Acc*
**DSC data**	**PM**	***C***_***0***_	0.84 (0.07)	0.74 (0.08)	0.78 (0.06)	0.81 (0.07)	0.79 (0.05)	0.65 (0.05)
		***CC***	0.29 (0.13)	0.88 (0.04)	0.38 (0.15)	0.84 (0.03)	0.59 (0.07)	
		***LC***	0.55 (0.12)	0.82 (0.05)	0.55 (0.09)	0.82 (0.04)	0.68 (0.06)	
	**KNN**	***C***_***0***_	0.97 (0.03)	0.91 (0.06)	0.92 (0.04)	0.96 (0.04)	0.94 (0.03)	0.80 (0.04)
		***CC***	0.42 (0.15)	0.95 (0.03)	0.70 (0.17)	0.87 (0.03)	0.69 (0.07)	
		***LC***	0.77 (0.10)	0.84 (0.05)	0.66 (0.07)	0.90 (0.04)	0.81 (0.05)	
	**DTW-KNN**	***C***_***0***_	0.95 (0.04)	0.91 (0.05)	0.92 (0.04)	0.95 (0.04)	0.93 (0.03)	0.80 (0.04)
		***CC***	0.35 (0.13)	0.96 (0.03)	0.71 (0.18)	0.86 (0.02)	0.66 (0.07)	
		***LC***	0.81 (0.09)	0.82 (0.05)	0.64 (0.07)	0.92 (0.04)	0.81 (0.05)	
**DSC and demographic data**	**PM**	***C***_***0***_	0.86 (0.07)	0.74 (0.08)	0.78 (0.06)	0.83 (0.07)	0.80 (0.05)	0.65 (0.05)
		***CC***	0.23 (0.12)	0.90 (0.04)	0.35 (0.17)	0.83 (0.02)	0.56 (0.06)	
		***LC***	0.56 (0.13)	0.80 (0.06)	0.53 (0.09)	0.82 (0.04)	0.68 (0.07)	
	**KNN**	***C***_***0***_	0.90 (0.06)	0.78 (0.08)	0.81 (0.05)	0.88 (0.06)	0.84 (0.04)	0.75 (0.04)
		***CC***	0.30 (0.12)	0.96 (0.03)	0.65 (0.21)	0.85 (0.02)	0.63 (0.06)	
		***LC***	0.80 (0.10)	0.85 (0.06)	0.70 (0.08)	0.92 (0.04)	0.83 (0.05)	
	**DTW-KNN**	***C***_***0***_	0.98 (0.02)	0.72 (0.09)	0.79 (0.05)	0.98 (0.03)	0.85 (0.05)	0.75 (0.05)
		***CC***	0.50 (0.16)	0.87 (0.05)	0.50 (0.14)	0.88 (0.04)	0.69 (0.08)	
		***LC***	0.44 (0.15)	0.96 (0.03)	0.83 (0.13)	0.81 (0.04)	0.70 (0.07)	

**Note**: The accuracy measures are denoted by sensitivity (*Sens*), specificity (*Spec*), positive predictive value (*PPV*), negative predictive value (*NPV*), balanced accuracy (*Bal Acc*) and accuracy (*Acc*). The groups are denoted by normal/ control (*C*_*0*_), cervical cancer (*CC*) and lung cancer (*LC*). The methods of classifications used are our parametric or proposed method (PM), KNN and DTW-KNN. Mean values of accuracy measures are shown with standard deviation in parentheses. Mean values less than 50 are shaded in red, values 50–84 are shaded in grey and values greater than or equal to 85 are shaded in green.

## Discussion

Over the last 10 years, DSC has been applied to the analysis of clinical samples in multiple disease settings [1–16, 21, 30]. It is hypothesized that DSC thermograms reflect alterations to plasma proteins in the disease state which affect the plasma denaturation profile. Although the mechanism underlying the observed DSC changes has not yet been reported, multiple reports demonstrate the potential utility of this approach for the detection and discrimination of disease [1–16, 21, 28, 30]. Extraction of diagnostic information from DSC thermogram data remains a challenging area with many different approaches having been applied [3, 8, 15–18]. We have previously reported the development of a parametric method for the analysis of DSC plasma thermogram data which provided superior performance compared with the established nonparametric method I-RELIEF. In this paper, we describe an extension of our previous work for multi-group classification and the inclusion of demographic data, which has not been reported in the literature.

In developing our approach we applied different transformation methods and determined that the logarithmic transformation (*H*_1_) was the optimal transformation based on the normality test at each temperature point (Tables A-C in S4 File) and gave the best separation between the normal and cancer groups based on principal component analysis (S1 Fig). We tested multiple models for both two-group and three-group classification. We tested models with 2- to 6- degree polynomials and found the best results with a 4-degree polynomial. We then applied the Akaike information criterion in a stepwise algorithm to determine the optimal set of explanatory variables in each model by selecting the best performing models with the lowest AIC values (Table 3).

Table 4 compares the performance of our parametric PM approach with two commonly used classification approaches, the non-parametric approach KNN and the semi-parametric approach DTW-KNN. The PM performed well for the two-group classification of normal versus cancer (accuracy 0.85–0.86; balanced accuracy 0.86–0.89) with high specificity (0.90–0.94) and moderate sensitivity (0.81–0.83). Classification of lung cancer versus cervical cancer is more challenging (accuracy 0.59; balanced accuracy 0.58) with low specificity (0.62) and sensitivity (0.54). The addition of demographic data to the model does not affect classification performance. Performance of PM for three-group classification (Table 5) is moderate (accuracy 0.65; balanced accuracy 0.59–0.79) with specificity remaining quite high (0.74–0.88) but with much lower sensitivity (0.29–0.84). Similarly, for two-group classification, model performance is not affected by the inclusion of demographic data (accuracy 0.65; balanced accuracy 0.56–0.80) with only small changes in sensitivity (decreases from 0.29 to 0.23 for cervical cancer and increases from 0.55 to 0.56 for lung cancer and 0.84 to 0.86 for normal).

Comparing the performance of PM to KNN and DTW-KNN for two-group classification, the specificity of the PM method is generally higher or the same as KNN and DTW-KNN but the sensitivity is lower resulting in slightly higher accuracy and balanced accuracy for KNN and DTW-KNN. With the inclusion of demographic data, the performance of KNN and DTW-KNN is lower whereas similar performance is observed for PM, hence the all three approaches have similar performance for two-group classification with demographic data. All three methods struggle with the separation of lung cancer from cervical cancer. Similarly, for three-group classification without demographic data, overall performance of PM (accuracy 0.65; balanced accuracy 0.59–0.79) is lower than KNN and DTW-KNN (accuracy 0.80; balanced accuracy 0.66–0.94). Inclusion of demographic data decreased the performance of KNN and DTW-KNN but performance of PM was similar thus making all three methods comparable. Overall, the class-wise classification results obtained from the three-group classification method shows that the normal samples are predicted more accurately as compared to cervical cancer and lung cancer samples.

One disadvantage of the KNN and DTW-KNN methods are the risk of the overfitting which can be observed from our results when the inclusion of demographic data decreased accuracy of both methods. This shows the KNN and DTW-KNN methods are sensitive to inclusion of additional covariates. The use of fewer parameters with the PM resulted in lower performance for models with no demographic data but higher performance when demographic data was included. This is of great interest in the further development of the PM where classification performance can be increased by the inclusion of additional demographic or clinical parameters as well as by using a greater number of quantile points and provides a substantial advantage over methods such as KNN and DTW-KNN. Alternative approaches such as support vector machine (SVM) methods [31–34] are of interest to the authors and may provide utility in analyzing thermogram data. In this study, we have correlated (time series) data with unequal numbers of samples in each clinical group. To apply SVM methods on time series data, there are some limitations for more than two groups. For this reason, we did not apply SVM methods in this study, but future work will consider the development of alternative forms of SVM methods that will be more appropriate for correlated data.

## Conclusions

In the present article, we have compared three methods (PM, KNN and DTW-KNN) using DSC and demographic data for classifying normal and cancer samples. We have developed and implemented the various steps of two-group and three-group classification methods using three different approaches using the statistical analysis program R. Future work will address the development of the PM for the classification of DSC datasets in other disease settings and the development of a classification prediction algorithm using DSC reference datasets.

## Supporting information

S1 Fig Principal component analysis. Principal component analysis biplot using (A) the original data (*H*) and (B) log transformed data (*H*_1_).
(TIF)

S1 File DSC thermogram data and demographic / clinical data for all samples included in this study. This file contains the DSC data and demographic / clinical data for all subjects within each clinical group (97 normal / control, 35 cervical cancer, 54 lung cancer). The file contains one worksheet for the DSC data and one worksheet for the demographic / clinical data for each of the three groups (six worksheets in total). The first row of the DSC data worksheet contains the column labels. The first column is labeled “Temperature” and contains the temperature values (in degrees Celsius) for each heat capacity measurement (451 data points between 45 °C and 90 °C at 0.1 °C intervals). Subsequent columns are labeled with a unique identifier for each subject and contain heat capacity data at each temperature point for a given subject. Subject identifiers starting with the letter L indicate lung cancer patients, the letter C indicates cervical cancer patients and N indicate normal / control subjects. The first row of the demographic / clinical worksheets contains the column labels indicating the sample ID (unique subject identifier) and relevant demographic / clinical variables for each group. The subsequent rows in the worksheet contain demographic / clinical data for a unique subject.
(XLSX)

S2 File Description of accuracy measures for two-group and three-group classification. (DOCX)

S3 File Description of the dynamic time warping (DTW) method. Flowchart of the dynamic time warping method (Figure A).
(DOCX)

S4 File Results of the normality test for different data transformations of the combined data for each pairwise combination of groups. Results of the normality test showing p-values at different temperature points using different data transformations for Normal vs. Cervical Cancer (Table A). Results of the normality test showing p-values at different temperature points using different transformations for Normal vs. Lung Cancer (Table B). Results of the normality test showing p-values at different temperature points using different transformations for Cervical Cancer vs. Lung Cancer (Table C).
(DOCX)

S5 File Results showing the effect of age on the thermogram profile for normal / control subjects. Summary of age for the normal group using a median cut-off of 36 years (Table A). The composite line plot and error bar plot (Figure A). (1) Composite line plot of HC values at each temperature data point for 49 normal samples with age ≤ 36 years (green) and 48 normal samples with age > 36 years (orange). (2) Composite error bar plot of HC values at each temperature data point for the two groups: normal samples with age ≤ 36 years (green) and normal samples with age > 36 years (orange). The circles represent mean values and the error bars represent the 95% confidence interval.
(DOCX)

S6 File The R functions related to the algorithms. Unzip/extract the file to get two R script files: “Classification_DSC.R” and “source.DSC.R”. Use “Classification_DSC.R” to obtain various outputs.
(ZIP)

## Abbreviations

Acc: Accuracy
AIC: Akaike information criterion
Bal Acc: Balanced accuracy
C_0_: Control
CC: Cervical cancer
DSC: Differential scanning calorimetry
DTW: Dynamic time warping
HC: Heat capacity
KNN: k-nearest neighbors
LC: Lung cancer
NPV: Negative predictive value
PPV: Positive predictive value
Sens: Sensitivity
Spec: Specificity
SVM: Support vector machine
