# Supplementary figures and images for: Multi-group diagnostic classification of high-dimensional data using differential scanning calorimetry plasma thermograms
Source: PLoS One. 2019 Aug 20;14(8):e0220765. doi: 10.1371/journal.pone.0220765 (PMC6701772; doi:10.1371/journal.pone.0220765)

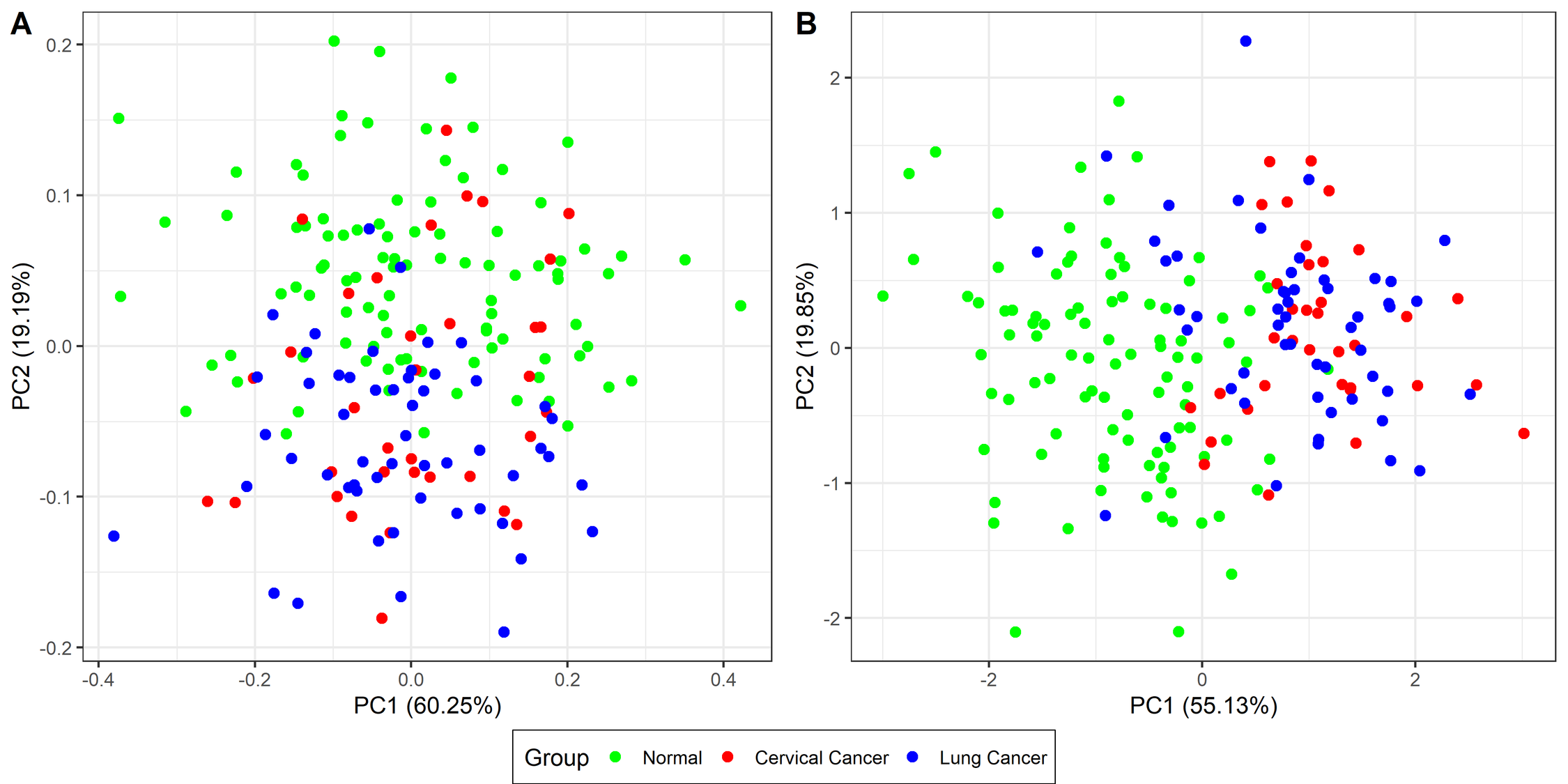

Supplement: S1 Fig — Principal component analysis biplot using (A) the original data (H) and (B) log transformed data (H1). (TIF) [file pone.0220765.s001.tif]
