# Supplementary material for: Multi-group diagnostic classification of high-dimensional data using differential scanning calorimetry plasma thermograms
Source: PLoS One. 2019 Aug 20;14(8):e0220765. doi: 10.1371/journal.pone.0220765 (PMC6701772; doi:10.1371/journal.pone.0220765)
Supplement: S2 File — (DOCX) [file pone.0220765.s003.docx]

**Accuracy Measures for two-group classification**

The confusion matrix for two-group classification is given below:

|  |  | **True Class** | |
| --- | --- | --- | --- |
|  |  | **Class 1** | **Class 2** |
| **Predicted Class** | **Class 1** | A | B |
|  | **Class 2** | C | D |

Accuracy measures are given below:

$$Sensitivity=\frac{A}{A+C}$$

$$Specificity=\frac{D}{B+D}$$

$$Prevalence=\frac{A+C}{A+B+C+D}$$

$$PPV=\frac{Sensitivity\times Prevalence}{\left( Sensitivity\times Prevalence \right)+\left( 1-Specificity \right)\times\left( 1-Prevalence \right)}$$

$$NPV=\frac{Specificity\times\left( 1-Prevalence \right)}{\left( 1-Sensitivity \right)\times Prevalence+Specificity\times\left( 1-Prevalence \right)}$$

$$Balanced Accuracy=\frac{Sensitivity+Specificity}{2}$$

$$Accuracy=\frac{A+D}{A+B+C+D}$$

**Accuracy Measures for three-group classification**

When there are three classes, the confusion matrix is calculated for a set of one-versus-all. There are three confusion matrices – (i) class 1 vs. (class 2 + class 3), (i) class 2 vs. (class 1 + class 3), (i) class 3 vs. (class 1 + class 2). Therefore, the result consists of class wise accuracy measures (sensitivity, specificity, PPV, NPV, prevalence, and balanced accuracy). The confusion matrix for three-group classification is given below:

|  |  | **True Class** | | |
| --- | --- | --- | --- | --- |
|  |  | **Class 1** | **Class 2** | **Class 3** |
| **Predicted Class** | **Class 1** | A | B | C |
|  | **Class 2** | D | E | F |
|  | **Class 3** | G | H | I |

The accuracy for three-group classification is given by

$$Accuracy=\frac{A+E+I}{A+B+C+D+E+F+G+H+I}$$
