# Supplementary material for: Multi-group diagnostic classification of high-dimensional data using differential scanning calorimetry plasma thermograms
Source: PLoS One. 2019 Aug 20;14(8):e0220765. doi: 10.1371/journal.pone.0220765 (PMC6701772; doi:10.1371/journal.pone.0220765)
Supplement: S3 File — Flowchart of the dynamic time warping method (Figure A). (DOCX) [file pone.0220765.s004.docx]

**Dynamic time warping (DTW) method**

The DTW algorithm is a sequence alignment algorithm that can be used to align two or more temporal series to facilitate quantifying similarity. A pseudo-distance metric, DTW, can be used to quantify the similarity between the series to construct a classifier. The advantage of this methodology is that it manipulates the time domain of samples to better facilitate pattern detection. A flowchart for optimal alignment of two series is shown below in Figure A:


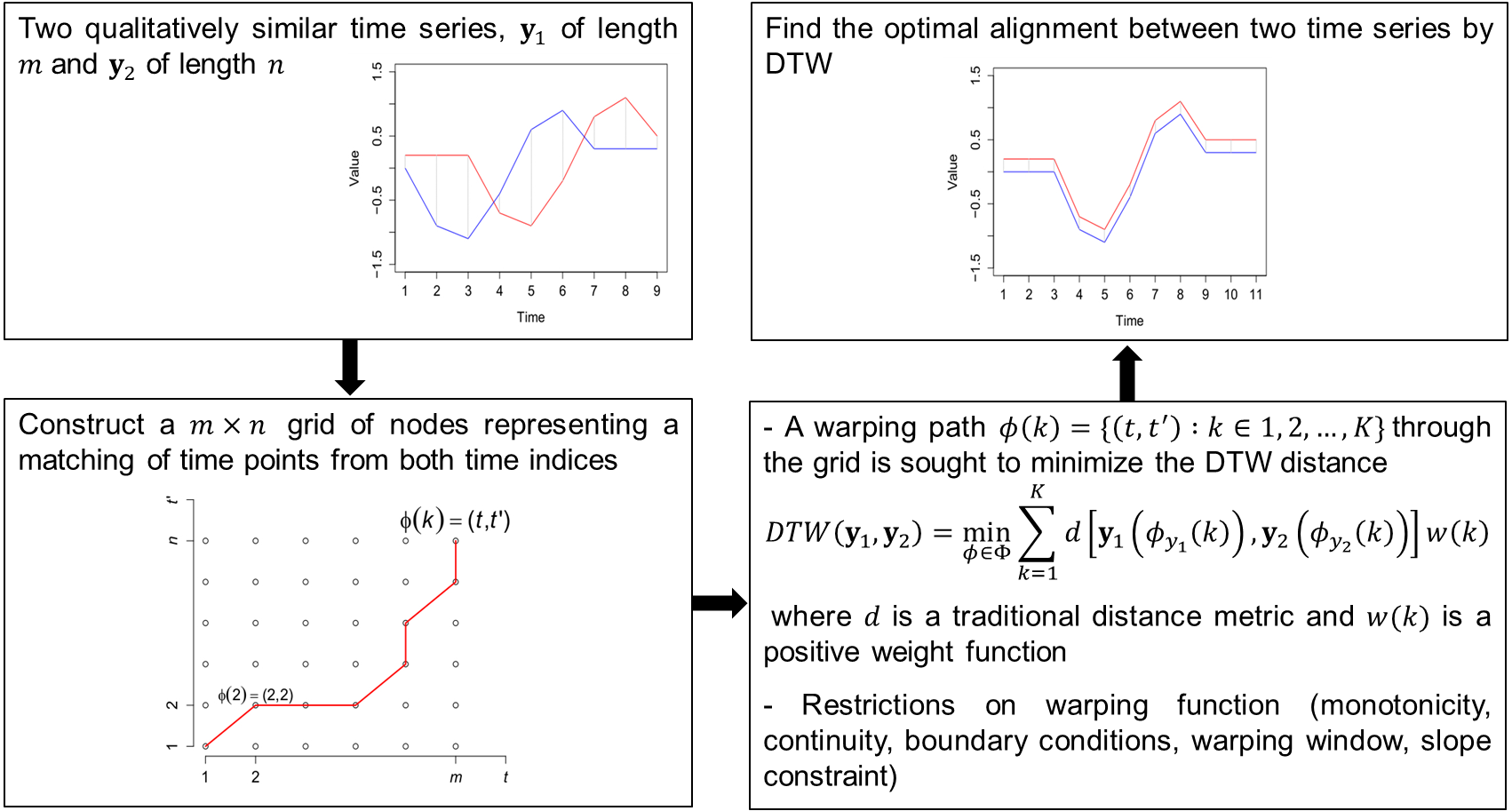


**Figure A.** Flowchart of the dynamic time warping method.
