# Supplementary material for: Multi-group diagnostic classification of high-dimensional data using differential scanning calorimetry plasma thermograms
Source: PLoS One. 2019 Aug 20;14(8):e0220765. doi: 10.1371/journal.pone.0220765 (PMC6701772; doi:10.1371/journal.pone.0220765)
Supplement: S4 File — Results of the normality test showing p-values at different temperature points using different data transformations for Normal vs. Cervical Cancer (Table A). Results of the normality test showing p-values at different temperature points using different transformations for Normal vs. Lung Cancer (Table B). Results of the normality test showing p-values at different temperature points using different transformations for Cervical Cancer vs. Lung Cancer (Table C). (DOCX) [file pone.0220765.s005.docx]

**Table A.** Results of the normality test showing p-values at different temperature points using different data transformations for Normal vs. Cervical Cancer

| **Temperature (°C)** | ***H*** | ***H_1_*** | ***H_2_*** | ***H_3_*** | ***H_4_*** |
| --- | --- | --- | --- | --- | --- |
| 48 | 0 | 0.14 | 0.15 | 0 | 0 |
| 49 | 0 | 0.01 | 0 | 0 | 0 |
| 50 | 0 | 0 | 0 | 0 | 0 |
| 51 | 0 | 0.03 | 0.03 | 0 | 0 |
| 52 | 0 | 0.27 | 0.25 | 0 | 0 |
| 53 | 0 | 0.57 | 0.58 | 0 | 0 |
| 54 | 0 | 0.51 | 0.51 | 0 | 0 |
| 55 | 0 | 0.73 | 0.72 | 0 | 0 |
| 56 | 0 | 0.42 | 0.39 | 0 | 0 |
| 57 | 0 | 0.65 | 0.6 | 0 | 0 |
| 58 | 0 | 0.25 | 0.21 | 0 | 0 |
| 59 | 0 | 0.06 | 0.05 | 0 | 0 |
| 60 | 0 | 0.01 | 0.01 | 0 | 0 |
| 61 | 0 | 0.27 | 0.12 | 0 | 0 |
| 62 | 0.03 | 0.27 | 0.73 | 0.04 | 0.09 |
| 63 | 0.79 | 0 | 0.02 | 0.75 | 0.57 |
| 64 | 0.02 | 0 | 0 | 0.01 | 0.01 |
| 65 | 0.42 | 0 | 0.02 | 0.38 | 0.28 |
| 66 | 0.09 | 0.52 | 0.36 | 0.1 | 0.13 |
| 67 | 0.02 | 0.23 | 0.13 | 0.02 | 0.03 |
| 68 | 0.06 | 0.57 | 0.36 | 0.07 | 0.1 |
| 69 | 0.31 | 0.88 | 0.8 | 0.34 | 0.43 |
| 70 | 0.52 | 0.83 | 0.86 | 0.56 | 0.66 |
| 71 | 0.43 | 0.79 | 0.84 | 0.47 | 0.57 |
| 72 | 0.29 | 0.94 | 0.94 | 0.32 | 0.42 |
| 73 | 0.03 | 0.96 | 0.85 | 0.04 | 0.06 |
| 74 | 0 | 0.69 | 0.33 | 0 | 0 |
| 75 | 0 | 0.13 | 0.04 | 0 | 0 |
| 76 | 0 | 0.06 | 0.02 | 0 | 0 |
| 77 | 0 | 0.05 | 0.02 | 0 | 0 |
| 78 | 0 | 0.19 | 0.11 | 0 | 0 |
| 79 | 0.03 | 0.31 | 0.4 | 0.03 | 0.03 |
| 80 | 0.53 | 0 | 0 | 0.53 | 0.53 |
| **Mean** | 0.11 | 0.34 | 0.32 | 0.11 | 0.12 |
| **No. of *P* > 0.05** | 9 | 24 | 21 | 9 | 11 |
| **% of *P* > 0.05** | 27.27 | **72.73** | 63.64 | 27.27 | 33.33 |

Legend: $H_{1}=\log\left( H \right), H_{2}=logit\left( H/{0.5} \right), H_{3}=\frac{e^{H}}{1+e^{H}}, H_{4}=\frac{e^{2H}}{1+e^{2H}}$

The results shows that the transformation$H_{1}$ is the best for two-group classification of Normal vs. Cervical Cancer.

**Table B.** Results of the normality test showing p-values at different temperature points using different data transformations for Normal vs. Lung Cancer

| **Temperature (°C)** | ***H*** | ***H_1_*** | ***H_2_*** | ***H_3_*** | ***H_4_*** |
| --- | --- | --- | --- | --- | --- |
| 48 | 0 | 0.01 | 0.01 | 0 | 0 |
| 49 | 0 | 0.02 | 0.01 | 0 | 0 |
| 50 | 0 | 0 | 0 | 0 | 0 |
| 51 | 0 | 0.02 | 0.02 | 0 | 0 |
| 52 | 0 | 0.18 | 0.18 | 0 | 0 |
| 53 | 0.01 | 0.3 | 0.32 | 0.01 | 0.01 |
| 54 | 0 | 0.69 | 0.71 | 0 | 0 |
| 55 | 0.03 | 0.41 | 0.43 | 0.03 | 0.03 |
| 56 | 0.01 | 0.44 | 0.45 | 0.01 | 0.01 |
| 57 | 0.01 | 0.23 | 0.24 | 0.01 | 0.01 |
| 58 | 0.01 | 0.2 | 0.21 | 0.01 | 0.01 |
| 59 | 0 | 0.38 | 0.36 | 0 | 0 |
| 60 | 0 | 0.09 | 0.04 | 0 | 0 |
| 61 | 0 | 0.4 | 0.12 | 0 | 0 |
| 62 | 0.02 | 0.24 | 0.89 | 0.03 | 0.08 |
| 63 | 0.92 | 0 | 0.09 | 0.93 | 0.92 |
| 64 | 0.17 | 0 | 0 | 0.13 | 0.06 |
| 65 | 0 | 0 | 0 | 0 | 0 |
| 66 | 0.79 | 0.42 | 0.7 | 0.8 | 0.83 |
| 67 | 0.36 | 0.94 | 0.88 | 0.39 | 0.48 |
| 68 | 0.26 | 0.92 | 0.84 | 0.3 | 0.39 |
| 69 | 0.39 | 0.89 | 0.88 | 0.44 | 0.56 |
| 70 | 0.51 | 0.92 | 0.94 | 0.56 | 0.69 |
| 71 | 0.28 | 0.85 | 0.85 | 0.32 | 0.43 |
| 72 | 0.11 | 0.93 | 0.88 | 0.13 | 0.19 |
| 73 | 0.01 | 0.86 | 0.56 | 0.01 | 0.01 |
| 74 | 0 | 0.35 | 0.1 | 0 | 0 |
| 75 | 0 | 0.06 | 0.01 | 0 | 0 |
| 76 | 0 | 0.02 | 0 | 0 | 0 |
| 77 | 0 | 0.04 | 0.01 | 0 | 0 |
| 78 | 0 | 0.19 | 0.1 | 0 | 0 |
| 79 | 0 | 0.67 | 0.7 | 0 | 0 |
| 80 | 0.24 | 0 | 0 | 0.24 | 0.25 |
| **Mean** | 0.13 | 0.35 | 0.35 | 0.13 | 0.15 |
| **No. of *P* > 0.05** | 10 | 23 | 22 | 10 | 11 |
| **% of *P* > 0.05** | 30.3 | **69.7** | 66.67 | 30.3 | 33.33 |

Legend: $H_{1}=\log\left( H \right), H_{2}=logit\left( H/{0.5} \right), H_{3}=\frac{e^{H}}{1+e^{H}}, H_{4}=\frac{e^{2H}}{1+e^{2H}}$

The results shows that the transformation$H_{1}$ is the best for two-group classification of Normal vs. Lung Cancer.

**Table C.** Results of the normality test showing p-values at different temperature points using different data transformations for Cervical Cancer vs. Lung Cancer

| **Temperature (°C)** | ***H*** | ***H_1_*** | ***H_2_*** | ***H_3_*** | ***H_4_*** |
| --- | --- | --- | --- | --- | --- |
| 48 | 0.41 | 0 | 0 | 0.41 | 0.41 |
| 49 | 0.55 | 0.08 | 0.09 | 0.55 | 0.55 |
| 50 | 0.14 | 0.16 | 0.18 | 0.14 | 0.14 |
| 51 | 0.15 | 0.05 | 0.06 | 0.15 | 0.15 |
| 52 | 0.46 | 0.03 | 0.04 | 0.46 | 0.46 |
| 53 | 0.23 | 0.06 | 0.06 | 0.23 | 0.23 |
| 54 | 0.02 | 0.03 | 0.03 | 0.02 | 0.02 |
| 55 | 0.02 | 0.11 | 0.11 | 0.02 | 0.02 |
| 56 | 0 | 0.16 | 0.16 | 0 | 0 |
| 57 | 0.03 | 1 | 1 | 0.04 | 0.04 |
| 58 | 0.12 | 0.97 | 0.97 | 0.12 | 0.12 |
| 59 | 0.03 | 0.43 | 0.42 | 0.03 | 0.03 |
| 60 | 0 | 0.41 | 0.28 | 0 | 0 |
| 61 | 0.08 | 0.33 | 0.56 | 0.08 | 0.11 |
| 62 | 0.07 | 0.08 | 0.31 | 0.09 | 0.13 |
| 63 | 0.18 | 0 | 0.01 | 0.18 | 0.17 |
| 64 | 0.81 | 0 | 0.03 | 0.78 | 0.68 |
| 65 | 0.48 | 0 | 0.02 | 0.44 | 0.33 |
| 66 | 0.28 | 0.55 | 0.52 | 0.3 | 0.33 |
| 67 | 0.14 | 0.13 | 0.15 | 0.14 | 0.14 |
| 68 | 0.01 | 0.01 | 0.01 | 0.01 | 0.01 |
| 69 | 0.24 | 0.09 | 0.15 | 0.24 | 0.22 |
| 70 | 0.75 | 0.36 | 0.56 | 0.75 | 0.74 |
| 71 | 0.72 | 0.5 | 0.68 | 0.73 | 0.75 |
| 72 | 0.27 | 0.48 | 0.54 | 0.28 | 0.33 |
| 73 | 0.05 | 0.57 | 0.48 | 0.05 | 0.07 |
| 74 | 0 | 0.32 | 0.18 | 0 | 0 |
| 75 | 0 | 0.18 | 0.08 | 0 | 0 |
| 76 | 0 | 0.23 | 0.14 | 0 | 0 |
| 77 | 0.01 | 0.78 | 0.66 | 0.01 | 0.01 |
| 78 | 0.01 | 0.74 | 0.65 | 0.01 | 0.01 |
| 79 | 0 | 0.38 | 0.33 | 0 | 0 |
| 80 | 0 | 0.74 | 0.7 | 0 | 0 |
| **Mean** | 0.19 | 0.3 | 0.31 | 0.19 | 0.19 |
| **No. of *P* > 0.05** | 18 | 25 | 26 | 19 | 19 |
| **% of *P* > 0.05** | 54.55 | 75.76 | **78.79** | 57.58 | 57.58 |

Legend: $H_{1}=\log\left( H \right), H_{2}=logit\left( H/{0.5} \right), H_{3}=\frac{e^{H}}{1+e^{H}}, H_{4}=\frac{e^{2H}}{1+e^{2H}}$

The results shows that the transformation$H_{2}$ is the best two-group classification of Cervical Cancer vs. Lung Cancer.
