# Supplementary material for: Multi-group diagnostic classification of high-dimensional data using differential scanning calorimetry plasma thermograms
Source: PLoS One. 2019 Aug 20;14(8):e0220765. doi: 10.1371/journal.pone.0220765 (PMC6701772; doi:10.1371/journal.pone.0220765)
Supplement: S5 File — Summary of age for the normal group using a median cut-off of 36 years (Table A). The composite line plot and error bar plot (Figure A). (1) Composite line plot of HC values at each temperature data point for 49 normal samples with age ≤ 36 years (green) and 48 normal samples with age > 36 years (orange). (2) Composite error bar plot of HC values at each temperature data point for the two groups: normal samples with age ≤ 36 years (green) and normal samples with age > 36 years (orange). The circles represent mean values and the error bars represent the 95% confidence interval. (DOCX) [file pone.0220765.s006.docx]

**Effect of age on the thermogram profile**

We divided the normal group into two groups using a median cut-off of 36 years: (1) Age ≤ 36 years and (2) Age > 36 years. The summary of age for the two groups is given below:

**Table A.** Summary of age for the normal group using a median cut-off of 36 years

|  | **Age** ≤ **36 years** | **Age > 36 years** |
| --- | --- | --- |
| **Number of samples** | 49 | 48 |
| **Mean** | 26.3 | 45.5 |
| **Median** | 24 | 46 |
| **Standard deviation** | 5.4 | 6.1 |

We compared the DSC thermograms for two groups as shown below in Fig A. We found that the patterns are similar for the two groups.


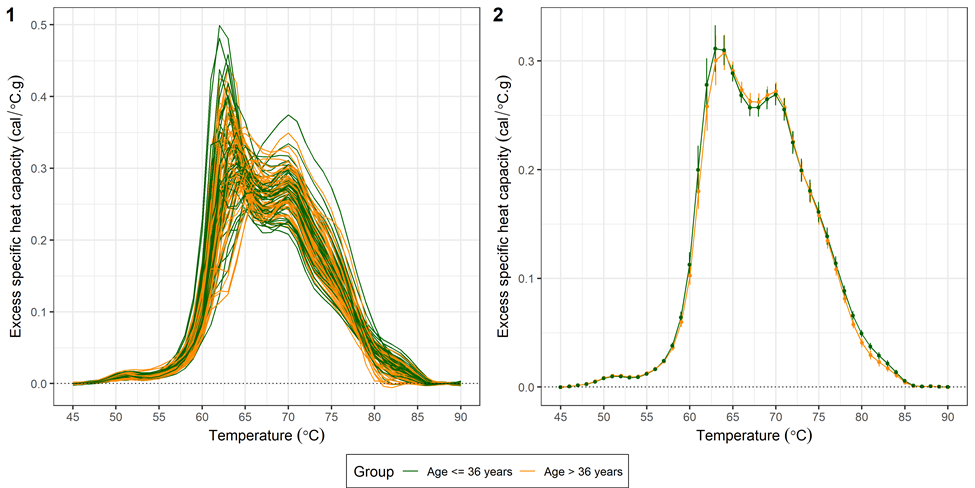


**Fig A.** **The composite line plot and error bar plot.** (1) Composite line plot of HC values at each temperature data point for 49 normal samples with age ≤ 36 years (green) and 48 normal samples with age > 36 years (orange). (2) Composite error bar plot of HC values at each temperature data point for the two groups: normal samples with age ≤ 36 years (green) and normal samples with age > 36 years (orange). The circles represent mean values and the error bars represent the 95% confidence interval.

Furthermore, we compared the two groups at each temperature point and tested for significance using the two-sample t-test based on DSC data on the logarithmic scale. The test was found to be significant (p-value < 0.05) for only three temperature points (78 ^o^C, 79 ^o^C and 80 ^o^C) out of a total of 33 points. Adjusting the p-values for multiple comparisons using the “bonferroni” method, we found the test to be significant at only one temperature point (79 ^o^C).

The effect of age is not significant in any of the models given in Table 3. The variable “age” has dropped out during model selection for two-group and three-group classification.
